# Knowledge, attitudes and practices among people with chronic hepatitis B attending a hepatology clinic in Malaysia: A cross sectional study

**DOI:** 10.1186/1471-2458-12-601

**Published:** 2012-08-03

**Authors:** Rosmawati Mohamed, Chirk Jenn Ng, Wen Ting Tong, Suraya Zainol Abidin, Li Ping Wong, Wah Yun Low

**Affiliations:** 1 Department of Medicine, Faculty of Medicine, University of Malaya, Malaya, 50603, Kuala Lumpur, Malaysia; 2 Department of Primary Care Medicine, Faculty of Medicine, University of Malaya, Malaya, 50603, Kuala Lumpur, Malaysia; 3 Medical Education and Research Development Unit, Faculty of Medicine, University of Malaya, Malaya, 50603, Kuala Lumpur, Malaysia; 4 University of Aberdeen, Malaya, Kuala Lumpur, Malaysia; 5 Department of Social and Preventive Medicine, Faculty of Medicine, University of Malaya, Malaya, 50603, Kuala Lumpur, Malaysia; 6 Dean’s Office, Faculty of Medicine, University of Malaya, Malaya, 50603, Kuala Lumpur, Malaysia

**Keywords:** Hepatitis B, Knowledge, Attitude, Practice

## Abstract

**Background:**

Hepatitis B (HBV) is the leading cause of cirrhosis and hepatocellular carcinoma worldwide. This study assessed the knowledge, attitudes and practices of people with chronic HBV and the associated factors.

**Methods:**

This cross-sectional study was conducted at an outpatient adult hepatology clinic at a tertiary hospital in Kuala Lumpur. A self-administered questionnaire was administered on a one-to-one basis to assess knowledge, attitudes, and lifestyle practices of people with chronic HBV.

**Results:**

The response rate was 89% (n = 483/543). Participants had a mean age of 46.3 (±14.7) years and the mean duration of HBV from time of diagnosis was 12.2 (±8.8) years. The mean knowledge score was 12.57/20 (standard deviation: ±4.4, range: 0–19). Participants aged 30–39 years, with higher educational attainment, employed in professional jobs, longer duration of diagnosis and those without cirrhosis had significantly higher knowledge scores. Age, education level and duration of diagnosis were significant predictors of the knowledge score on standard multiple regression analysis. More than half of the participants were worried of spreading HBV infection to family and friends and worried since the diagnosis. A third of the participants (33.5%) were embarrassed to reveal their diagnosis to the public but most of them (93.6%) would inform their family. Those who reported feeling worried since their diagnosis were more likely to be middle-aged, of Malay ethnicity, have shorter duration of diagnosis of less than 10 years and have received therapy. About half of the participants (50.6%) did not share dining utensils and the majority (93.2%) believed that HBV can be transmitted by sharing of eating and drinking utensils. Older patients were significantly less likely to share utensils. Those who felt worried since diagnosis had significant higher knowledge of HBV.

**Conclusion:**

The findings highlight the stigma and misconceptions that still exist among the HBV patients. More patient and public education about HBV and its prevention are essential to increase awareness and to demystify the disease.

## Background

Hepatitis B (HBV) is the leading cause of cirrhosis and hepatocellular carcinoma worldwide [1,2]. Approximately 350 million people in the world are chronically infected with HBV [3-5] and an estimated three quarters of these individuals are from the Asia-Pacific region [4,6]. Despite the high prevalence of HBV infection amongst Asians, a number of studies have consistently shown a lack in general public knowledge of HBV infection in this population, especially with regard to the modes of transmission and complications of chronic HBV infection such as cirrhosis, liver failure and hepatocellular carcinoma [7-11].

Malaysia is one of the countries in Asia which has an intermediate level of chronic HBV endemicity with an estimated prevalence of 5% [12-14]. Despite this, there are only a few studies in Malaysia which have looked at how people with HBV infection view their illness and how it has affected their lives and those around them. A recent qualitative study in Malaysia revealed that patients who were newly diagnosed with HBV were anxious over their diagnoses. Factors that contributed to the patients’ anxiety included lack of background knowledge on HBV and the perceived stigma associated with the disease. They wanted their healthcare providers to give more information on HBV and its treatment options [15].

Patient’s knowledge of symptoms, severity, monitoring and treatment options are essential for appropriate self care, adherence to follow up, early recognition of red flag signs and seeking timely treatment [16]. A study which looked at health attitudes amongst patients with chronic HBV in North America found that 70% of Asian patients in the USA had never received treatment for chronic HBV and were less likely to report symptoms of their disease [17]. Another study amongst Asian/Pacific Islanders with chronic HBV in the United States revealed that 75% of the study population was unsure of how they acquired HBV while 41% claimed that they did not practice any prevention methods to avoid HBV transmission to their close contacts [18]. Misperceptions such as early stage of liver cancer would caused symptoms, HBV transmission through sharing of food or eating and drinking utensils and consuming seafood have been identified in patients with chronic HBV [18-20]. The misconception that severe liver disease due to hepatitis B would show symptoms may contribute to unwillingness of patients to take time to attend regular medical follow up [21], and this would cause deterioration of the disease condition if treatment is delayed.

By understanding patients’ knowledge, attitudes, and lifestyle practices following diagnoses, health providers can begin to provide patient-centered care and education, help patients and their family to better understand the illness and help them to lead as normal a lifestyle as possible. In addition, modifying patients’ attitudes and behavior could promote disease monitoring of HBV patients by their attending physicians in terms of enhancing adherence to follow up and, in turn, result in early detection and treatment of disease reactivation and complications. Therefore, this study aimed to determine the knowledge, attitudes and lifestyle practices of Malaysian patients who have been diagnosed with chronic HBV.

## Methods

### Study setting and participants

This survey was conducted from March 2007 to April 2008 in an adult outpatient hepatology clinic at the University of Malaya Medical Centre (UMMC), which is an urban tertiary hospital in Kuala Lumpur, Malaysia. Consecutive HBV patients attending the clinic were identified. A total of 543 chronic HBV patients were approached for participation. Out of the 543 patients, 60 were not willing to participate. Therefore, a total of 483 chronic HBV patients were included in the analysis.

Cirrhosis was diagnosed either histologically or clinically (radiological evidence of nodular or shrunken liver, or the presence of splenomegaly and/or signs of portal hypertension). The study was approved by the University of Malaya Medical Ethics Committee and written informed consents were obtained from all patients.

### Study instrument

Due to the multi-ethnic origins of the participants, the questionnaire was written in English, Malay and Mandarin. Trained research assistants and enumerators administered the questionnaires on a one-to-one basis and each interview took approximately 30 minutes to complete.

The questionnaire was developed based on literature review and consensus from the research team. A conceptual framework was developed to identify independent variables (e.g. the participant’s socio-demographic details, HBV disease profile, status of HBV treatment) which might influence the dependent variables (e.g. knowledge, attitudes and practices).

The knowledge section of the questionnaire tested on 3 aspects: (1) general knowledge of HBV (5 items); (2) symptoms of liver disease (5 items) and (3) modes of transmission (10 items). Participants’ attitudes towards HBV were also examined and they were asked to give their opinions on eight statements using a 5-point Likert scale (from 1 = strongly agree to 5 = strongly disagree). Two items were chosen to represent the overall attitude towards HBV, namely ‘worried of spreading HBV to family and friends’ and ‘worried ever since diagnosis’. Those who strongly agreed or agreed with the statements were considered to have negative attitudes while the rest were considered as having positive attitudes towards the disease. Participants’ lifestyle practices after being diagnosed with HBV were also examined.

### Data analyses

The data was analyzed using SPSS version 15. Descriptive analyses were used for the baseline socio-demographic and clinical data. The overall knowledge score is a summation of the 20 items whereby each correct response is given one point, thus giving it a maximum of 20 points. Bivariate analyses (student independent *t*-test, chi-square test and ANOVA) were conducted to test the associations between socio-demographic and clinical data with knowledge, negative attitudes and inappropriate practices that majority of the participants had. Significant predictors of knowledge, attitudes and practices were assessed using standard multiple regression and logistic regression. For logistic regression, ethnicity was grouped as Chinese and Non-Chinese. Malay, Indian and Other race were grouped to form ‘Non-Chinese’ as there was a very small number of participants grouped under ‘Indian’ and ‘Other’ ethnicity. The significance level was determined at p ≤ 0.05.

## Results

### Characteristics of survey sample

There were a total of 483 chronic HBV patients who were included in this study. The response rate for this study was 89%. Of these, 275 (56.9%) were male and 208 (43.1%) were female. By ethnicity, majority were Chinese (73.1%), followed by Malays (22.6%), Indians (2.5%) and others (1.9%) such as Bidayuh, Iban, Indonesian, Arab, and Thai. Participants had a mean age of 46.3 (±14.7) years and the majority of them (87.1%) have had at least secondary education. The mean duration of illness from time of diagnosis was 12.2 years (±8.8) and majority of them (77.2%) have never received any specific treatment for HBV. Evidence of cirrhosis was present in 15.4% of the participants.

### Knowledge of HBV

The mean knowledge score of the 20-item questions was 12.57 (±4.4). Almost half of the participants could not differentiate whether HBV is a viral or bacterial infection. Majority were aware that the clinical consequences of HBV infection include inflammation of the liver (81.6%), liver failure (78.5%) and liver cancer (85.1%). Only slightly more than half of the participants knew that tiredness (53.6%) and jaundice (55.3%) were symptoms of liver disease. However, less than half did not know that abdominal pain, abdominal distension and nausea and vomiting were symptoms of liver disease. Knowledge on modes of transmission was reasonably good and the majority achieved correct responses above 70th percentile. Nevertheless, only a small proportion of participants knew that the HBV virus could be transmitted through body piercing and tattooing (14.7%) but not via sharing of utensils (6.8%). (Table 1)

**Table 1:** Proportion of participants who had correct answers for knowledge items on Hepatitis B (n = 483)

**Hepatitis B knowledge (correct answer)**	**N (%)**
	**(n = 483)**
**General statements**	
Hepatitis B is a bacterial infection (No)	265 (55.0)
Hepatitis B is a viral infection (Yes)	303 (62.7)
Can cause chronic inflammation of liver (Yes)	394 (81.6)
Can cause liver failure (Yes)	379 (78.5)
Can cause liver cancer (Yes)	411 (85.1)
**Symptoms**	
Jaundice (Yes)	267 (55.3)
Tiredness (Yes)	259 (53.6)
Nausea and vomiting (Yes)	174 (36.0)
Abdominal discomfort or pain (Yes)	209 (43.3)
Abdominal distension (Yes)	190 (39.3)
**Modes of transmission**	
Sharing of needles (Yes)	412 (85.3)
Sexual intercourse (Yes)	383 (79.3)
Perinatal transmission (Yes)	405 (83.9)
Blood transfusion (Yes)	438 (90.7)
Contact with open wound (Yes)	378 (78.3)
Sharing eating and drinking utensils (No)	33 (6.8)
Sharing personal items-razors, toothbrushes (Yes)	371 (76.8)
Coughing and sneezing (No)	303 (62.7)
Body piercing and tattooing (Yes)	71 (14.7)
Casual contact (No)	424 (87.8)

Table 2 summarises the results of the bivariate analysis to detect associations between the mean knowledge score with socio-demographic and clinical characteristics of participants. The knowledge scores were significantly higher among participants aged 30–39 years with tertiary level education attainment, employed in professional jobs, had been diagnosed for more than 20 years and without cirrhosis. There were no significant difference in the mean knowledge score between gender, ethnicity and for ever received therapy for HBV infection.

**Table 2:** Bivariate analysis of the association between mean knowledge scores and socio-demographic and clinical characteristics of the study population (n = 483)

**Characteristic**	**Number**	**Mean (±sd)**	**Test value**	**p value**
**Age (years)**				
<30	78	12.63 (4.06)	F = 4.285	0.002*
30–39	84	13.75 (3.43)		
40–49	96	12.03 (5.03)		
50–59	134	13.03 (4.12)		
≥60	91	11.30 (4.78)		
**Gender**				
Male	275	12.37 (4.46)	t = −1.14	ns
Female	208	12.83 (4.29)		
**Ethnicity**				
Malay	109	12.82 (4.07)	F = 2.30	ns
Chinese	353	12.63 (4.39)		
Indian	12	10.17 (5.24)		
Others	9	10.11 (5.82)		
**Education level (Age)**				
Primary (7–12 years) and under	62	8.89 (4.69)	F = 42.38	≤0.001**
Secondary (13–17 years)	235	12.21(4.49)		
Tertiary (18 years and above)	186	14.24 (3.14)		
**Occupation**				
Professional	83	13.98 (3.81)	F = 7.26	≤0.001**
Skilled	41	10.32 (4.60)		
Unskilled	177	13.08 (4.09)		
Unemployed	119	12.26 (4.15)		
Housewife	63	11.30 (5.29)		
**Duration of diagnosis (years) (n = 478)**^**^**^				
<10	208	11.59 (4.70)	F=11.24	≤0.001**
10-19	161	13.01 (4.18)		
≥20	109	13.85 (3.43)		
**Ever received therapy (n=194)**^**#**^				
Yes	110	13.01 (4.21)	t=1.86	ns
No	84	11.82 (4.64)		
**Presence of Cirrhosis**				
Yes	74	11.16 (4.97)	t= 2.67	0.008*
No	409	12.82 (4.23)		

sd, standard deviation; ns, not significant; *significant different at p≤0.05; **significant different at p≤0.001; ^^^Missing data n=5; ^#^Applicable to those who were offered HBV therapy. Only those with significant liver disease and viral replication are offered treatment [22].

### Predictors of knowledge of HBV

The five variables that were identified as significant predictors of mean knowledge score (age, education level, occupation, duration of diagnosis and presence of cirrhosis) were further analysed by standard multiple regression. The results indicated that younger age group, tertiary education level and longer duration since diagnosis were significant predictors of higher knowledge score (F (5, 472) = 18.94, p≤ 0.001). The model explains 16.7% of the variance in the mean knowledge score (Table 3).

**Table 3:** Factors predictive of mean knowledge score in people with chronic Hepatitis B

**Variable**	**β**	**t**	**p value**	**R**	**R**^**2**^	**Adj R**^**2**^
**Age**	−0.045	−3.112	0.002*	0.409	0.167	0.159
**Duration of diagnosis**	0.138	6.081	≤0.001**			
**Education level**	2.301	5.746	≤0.001**			
**Occupation**	−0.285	−0.654	ns			
**Presence of Cirrhosis**	−0.878	−1.651	ns			

*significant different at p≤0.05; **significant different at p≤0.001.

### Attitudes towards HBV

**Figure 1:**
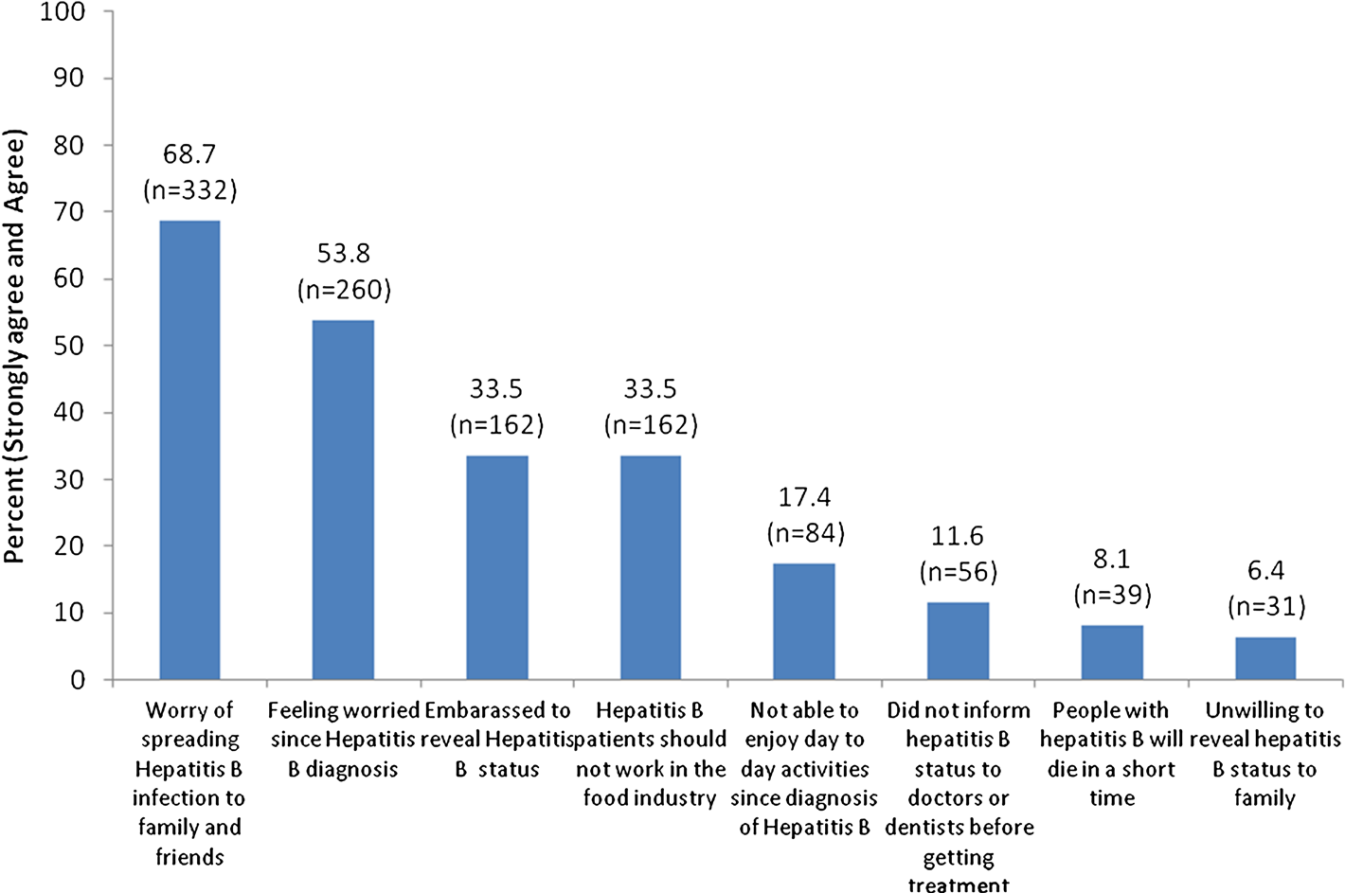
Attitudes towards HBV among people with chronic Hepatitis B (n = 483).

Figure 1 shows that more than half of the participants were worried about spreading HBV to their family and friends and slightly over half were worried ever since their diagnoses. An equal proportion of the participants were embarrassed to reveal their diagnosis to the public and were against HBV patients working in a food industry (33.5%), but majority would reveal their HBV status to their families. Over a quarter reported that they were unable to enjoy their daily activities since diagnosis of HBV. Only 11.6% of the participants did not disclose their HBV status to doctors or dentists, while 8% of them believed that people with HBV would die in a short time.

The two negative attitudes most reported (by more than half of the participants), namely, “worried about spreading HBV to family and friends” and “feeling worried ever since diagnosis” were further analysed in bivariate analyses to determine the associated socio-demographic and clinical variables (Table 4). The majority of the participants who reported feeling worried about spreading HBV to their family and friends were Malay, aged less than 30 years old and were diagnosed with HBV within a period of less than 10 years. Among those who reported worried ever since diagnosis were Malay and Indian, aged 30–39 years old, with HBV diagnosis of less than 10 years and who received HBV therapy.

**Table 4:** Bivariate analyses of the associations between characteristics of study population and attitudes and perceptions towards Hepatitis B (n=483)

**Characteristics**	**Negative attitudes and perceptions towards Hepatitis B**			
	**Worried ever since diagnosis (n=260)**		**Worried of spreading infection to family and friends (n= 332)**	
	**n (%)**	**p value**	**n (%)**	**p value**
**Age (years)**				
<30	46 (59.0)	0.001**	67 (85.9)	0.001**
30–39	59 (70.2)		64 (76.2)	
40–49	54 (56.3)		65 (67.7)	
50–59	64 (47.8)		87 (64.9)	
≥60	37 (40.7)		49 (53.8)	
**Gender**				
Male	148 (53.8)	ns	189 (68.7)	ns
Female	112 (53.8)		143 (68.8)	
**Ethnicity**				
Malay	73 (67.0)	0.01*	87 (79.8)	0.026*
Chinese	174 (49.3)		229 (64.9)	
Indian	8 (66.7)		9 (75.0)	
Others	5 (55.6)		7 (77.8)	
**Education level (Age)**				
Primary (7–12 years) and under	33 (53.2)	ns	37 (59.7)	ns
Secondary (13–17 years)	132 (56.2)		166 (70.6)	
Tertiary (18 years and above)	95 (51.1)		129 (69.4)	
**Occupation**				
Professional	44 (53.0)	ns	57 (68.7)	ns
Skilled	25 (61.0)		29 (70.7)	
Unskilled	104 (58.8)		132 (74.6)	
Unemployed	52 (43.7)		73 (61.3)	
Housewife	35 (55.6)		41 (65.1)	
**Duration of diagnosis**				
**(years) (n=478)**^**^**^	(n=258)^a^		(n=330)^c^	
<10	125 (60.1)	0.016*	157 (75.5)	0.012*
10 to 19	86 (53.4)		108 (67.1)	
≥20	47 (43.1)		65 (59.6)	
**Ever received therapy (n=194)**^**#**^	(n=104)^b^		(n=121)^d^	
Yes (n=110)	67 (60.9)	0.03*	73 (66.4)	ns
No (n=84)	37 (44.0)		48 (57.1)	
**Presence of Cirrhosis**				
Yes	32 (43.2)	ns	45 (60.8)	ns
No	228 (55.7)		287 (70.2)	

ns, not significant; *significant different at p≤0.05; **significant different at p≤0.001; ^^^Missing data n=5; ^#^Applicable to those who were offered HBV therapy. Only those with significant liver disease and viral replication are offered treatment [22]; ^a^missing data n=2; ^b^missing data n=156; ^c^missing data n=2; ^d^missing data n=211.

### Predictors of worry of spreading HBV infection to family and friends

Significant variables (age, ethnicity and duration of diagnosis) which were associated with worry of spreading infection to family and friends were further analysed using multiple logistic regression. This is to assess the impact of the variables on the likelihood that participants would feel worry of spreading HBV infection to family and friends. The full model were statistically significant at χ² (3, n = 478) = 25.1%, p ≤ 0.001. The model suggests that patients with increasing age were significantly more likely to be worry of spreading infection to family and friends (OR 1.027, 95% CI 1.012 to 1.042, p = 0.001)(Table 5).

**Table 5:** Logistic regression predicting worry of spreading infection to family and friends

**Predictor variable**	**B**	**SE**	**Wald**	**df**	**p value**	**OR**	**95% CI**	
							**Lower**	**Upper**
**Age (years)**	0.027	0.008	12.05	1	0.001**	1.027	1.012	1.042
**Duration of Diagnosis**	0.009	0.012	0.52	1	0.471	1.009	0.985	1.034
**Ethnicity**								
Non chinese	Reference							
Chinese	0.418	0.268	2.431	1	0.119	1.519	0.898	2.57
**Constant**	−2.498	0.381	42.91	1	0.00	0.82		

**significant different at p≤0.001.

### Predictors of worry ever since diagnosis of HBV

Multiple logistic regression was also performed with significant predictors (age, ethnicity, duration of diagnosis, and ever received HBV therapy) to determine the impact of the variables on the likelihood that HBV patients would feel worry ever since diagnosis of HBV. The full model were statistically significant at χ² (4, n = 189) = 13.2%, p ≤ 0.05. The model suggests that patients with older age were significantly more likely to be worried ever since diagnosis of HBV (OR 1.03, 95% CI 1.01 to 1.06, p = 0.007). Meanwhile, patients who received therapy for HBV were significantly less likely to be worried after diagnosis (Table 6).

**Table 6:** Logistic regression predicting worry ever since diagnosis

**Predictor variable**	**B**	**SE**	**Wald**	**df**	**p value**	**OR**	**95% CI**	
							**Lower**	**Upper**
**Age (years)**	0.032	0.012	7.26	1	0.007*	1.03	1.01	1.06
**Ethnicity**								
Non chinese	Reference							
Chinese	−0.023	0.386	0.004	1	0.952	0.977	0.459	2.082
**Duration of diagnosis**	−0.007	0.021	0.105	1	0.746	0.993	0.954	1.034
**Ever received therapy**	−0.705	0.312	5.094	1	0.024*	0.494	0.268	0.911
**Constant**	−1.198	0.584	4.208	1	0.04	0.302		

*significant different at p≤0.05.

### Lifestyle practices after HBV diagnosis

With respect to lifestyle practices after diagnosis, 57.3% made healthier food choices and 46.6% increased their exercise activity. Out of a total of 100 patients who reported having ever smoked, more than half (78.0%) have stopped or reduced smoking. Out of a total of 150 patients who reported ever drinking alcohol, majority (87.3%) reported they have stopped or reduced alcohol drinking.

For prevention of transmission, almost all the participants avoided sharing personal items such as razors and toothbrushes (98.3%) and did not engage in blood donation (99.0%). Most of them (91.7%) encouraged their immediate family members to undergo screening for HBV. However, half of the participants (50.6%) avoided sharing eating and drinking utensils with others (Table 7).

**Table 7:** Positive lifestyle practices among people with chronic Hepatitis B after diagnosis (n = 483)

**Lifestyle practices after diagnosis**	**N (%)**
Stopped smoking (n=100)^#^	78 (78%)
Reduced alcohol intake (n=150)^¥^	131 (87.3)
Increased exercise (n=292)^~^	225 (77.1)
Healthier food choice (n=289)^a^	277 (95.8)
Sharing eating and drinking utensils (n=480)^b^	237 (49.4)
Encouraged family members to undergo HBV screening	443 (91.7)
Did not engage in blood donation (n=477)^c^	472 (99.0)
Did not share personal items (razors, toothbrush) with others (n=481)^d^	473 (98.3)

^#^Applicable to those who smokes; ^¥^Applicable to those who consume alcohol; ^~^Applicable to those who exercise; ^a^missing data n=194; ^b^missing data n=3; ^c^missing data n=6; ^d^missing data n=2.

No significant associations between socio-demographic and clinical characteristics of participants and those who stopped or reduced smoking and did not increase exercise were shown, except for ethnicity, where there was a higher proportion of Indian who stopped or reduced alcohol intake. Participants of age group 60 years old or more, Indian and with cirrhosis were more likely to avoid sharing eating and drinking utensils (Table 8).

**Table 8:** Associations between lifestyle practices after diagnosis and characteristics of the study population (n=483)

**Characteristic**	**Stopped or reduced alcohol (n= 131)**		**Stopped or reduced smoking (n=78)**		**Not sharing eating and drinking utensils (n=243)**		**Did not increased exercise (n=67)**	
	**n (%)**	**p value**	**n (%)**	**p value**	**n (%)**	**p value**	**n (%)**	**p value**
**Age (years)**								
<30	14 (77.8)	ns	8 (61.5)	ns	33 (42.9)	≤0.001**	9 (22.0)	ns
30–39	25 (80.6)		18 (78.3)		31 (36.9)		13 (23.6)	
40–49	35 (89.7)		20 (74.1)		43 (45.3)		23 (32.4)	
50–59	34 (94.4)		18 (90.0)		74 (55.6)		14 (19.2)	
≥60	23 (88.5)		14 (82.4)		62 (68.1)		8 (15.4)	
**Gender**								
Male	95 (87.2)	ns	60 (78.9)	ns	141(51.5)	ns	38 (22.2)	ns
Female	36 (87.8)		18 (75.0)		102(49.5)		29 (24.0)	
**Ethnicity**								
Malay	11 (61.1)	0.002*	16 (69.6)	ns	46 (42.6)	0.038*	17 (27.9)	ns
Chinese	117(90.7)		60 (81.1)		186(52.8)		44 (20.4)	
Indian	3 (100)		2 (66.7)		9 (75.0)		3 (33.3)	
Others	0 (0)		0 (0)		2 (25.0)		3 (50.0)	
**Education level (Age)**								
Primary (7–12 years) and under	15 (88.2)	ns	12 (92.3)	ns	29 (46.8)	ns	8 (22.2)	ns
Secondary (13–17 years)	66 (84.6)		43 (76.8)		129(55.1)		37 (24.3)	
Tertiary (18 and above)	50 (90.9)		23 (74.2)		85 (46.2)		22 (21.2)	
**Occupation**								
Professional	32 (84.2)	ns	14 (60.9)	ns	43 (51.8)	ns	9 (16.7)	ns
Skilled	14 (87.5)		10 (90.9)		17 (41.5)		7 (29.2)	
Unskilled	52 (94.5)		32 (84.2)		82 (46.6)		28 (25.9)	
Unemployed	23 (79.3)		18 (81.8)		70 (59.3)		18 (26.1)	
Housewife	10 (83.3)		4 (66.7)		31 (50.0)		5 (13.5)	
**Duration of diagnosis (years) (n=478)**^**^**^					(n=241)^a^		(n=65)^a^	
<10	56 (84.8)	ns	35 (76.1)	ns	102(49.5)	ns	28 (21.9)	ns
10 to 19	42 (91.3)		23 (79.3)		81 (50.6)		22 (23.9)	
≥20	33 (89.2)		20 (83.3)		58 (53.2)		15 (21.7)	
**Ever received therapy(n=194)**^**#**^	(n=61)^b^		(n=47)^c^		(n=99)^d^		(n=35)^e^	
Yes (n=110)	38 (88.4)	ns	26 (89.7)	ns	59 (54.1)	ns	19 (26.0)	ns
No (n=84)	23 (85.2)		21 (77.8)		40 (47.6)		16 (28.6)	
**Presence of Cirrhosis**								
Yes	23 (92.0)	ns	16 (84.2)	ns	47 (63.5)	0.022*	11 (24.4)	ns
No	108 (86.4)		62 (76.5)		196(48.3)		56 (22.7)	

ns, not significant; *significant different at p≤0.05; **significant different at p≤0.001; ^^^Missing data n=5; ^#^Applicable to those who were offered HBV therapy. Only those with significant liver disease and viral replication are offered treatment [22]; ^a^missing data n=2; ^b^missing data n=70; ^c^missing data n=31; ^d^missing data n=144; ^e^missing data n=32.

### Predictors of sharing eating and drinking utensils

Age, ethnicity and presence of cirrhosis which were significant in the bivariate analyses were included in a multiple logistic regression model to determine the likelihood of HBV patients sharing eating and drinking utensils. The full model were statistically significant at *χ*² (3, n = 480) = 22.62%, p ≤ 0.001. The model suggests that older age was significantly less likely to share eating and drinking utensils (OR 0.97, 95% CI 0.96 to 0.99, p = ≤0.001)(Table 9).

**Table 9:** Logistic regression predicting sharing eating and drinking utensils

**Predictor variable**	**B**	**SE**	**Wald**	**df**	**p value**	**OR**	**95% CI**	
							**Lower**	**Upper**
**Age (years)**	−0.03	0.007	13.95	1	0.001**	0.97	0.96	0.99
**Ethnicity**								
Non Chinese	Reference							
Chinese	−0.08	0.22	0.13	1	0.718	0.92	0.6	1.42
**Presence of cirrhosis**								
No	Reference							
Yes	−0.39	0.27	2.19	1	0.14	0.67	0.39	1.14
**Constant**	1.30	0.33	15.78	1	0.000	3.64		

**significant different at p≤0.001.

### Associations between knowledge, attitudes and practices

No significant difference was found between patients’ HBV mean knowledge score with feeling worried of spreading HBV to family and friends. However, patients who felt worried ever since HBV diagnosis had significantly higher knowledge score compared to those who did not, t (483) = 2.3, p ≤ 0.05 (Table 10). There was no significant difference in participants’ knowledge score of HBV and lifestyle practices after diagnosis. Similarly, there were no associations between attitudes and lifestyle practices after HBV diagnosis (Table 11).

**Table 10:** Attitudes, lifestyle practices after diagnosis and overall mean knowledge scores (n=483)

**Attitude**	**Knowledge of HBV**		
	**n**	**Mean (±sd)**	**p value**
**Worried of spreading HBV to family and friends**			
Yes	332	12.8 (4.08)	ns
No	151	11.9 (4.96)	
**Feeling worried ever since diagnosis**			
Yes	260	12.9 (4.08)	
No	223	12.07 (4.68)	0.023*
**Lifestyle practice**			
**Stop or reduce smoking (n=100)**^**#**^			
Yes	78	12.2 (4.81)	ns
No	22	11.2 (5.95)	
**Stop or reduce alcohol (n=150)**^**¥**^			
Yes	131	12.9 (4.38)	ns
No	19	11.21 (5.78)	
**Sharing eating and drinking utensils (n=480)**^**a**^			
Yes	237	12.7 (4.51)	ns
No	243	12.5 (4.29)	
**Increasing exercise (n=292)**^**~**^			
Yes	225	13.19 (4.09)	ns
No	67	12.31 (4.64)	

sd, standard deviation; ns, not significant;*significant different at p≤0.05; ^#^Applicable to those who smokes; ^¥^Applicable to those who consume alcohol; ^~^Applicable to those who exercise; ^a^missing data n=3.

**Table 11:** Associations between attitudes and lifestyle practices of study population (n=483)

**Variable**	**Negative attitude and perception**					
	**Worried of spreading HBV to family and friends (n=332)**			**Worried ever since diagnosis (n=260)**		
**Lifestyle practice**	n	(%)	p value	n	(%)	p value
**Stop or reduced alcohol (n=150)**^**¥**^						
Yes	91	(91)	ns	74	(91.4)	ns
No	9	(9)		7	(8.6)	
**Stop or reduced smoking (n=100)**^**#**^						
Yes	55	(79.7)	ns	43	(81.1)	ns
No	14	(20.3)		10	(18.9)	
**Sharing eating and drinking utensils(n=480)**^**a**^						
Yes	165	(50)	ns	138	(53.3)	ns
No	165	(50)		121	(46.7)	
**Exercise regularly (n=292)**^**~**^						
Yes	164	(78.5)	ns	124	(74.7)	ns
No	45	(21.5)		42	(25.3)	

ns, not significant; ^#^Applicable to those who smokes; ^¥^Applicable to those who consume alcohol; ^~^Applicable to those who exercise; ^a^missing data n=3.

## Discussion

### Knowledge of HBV

This study found that there were gaps in the knowledge with regards to HBV among individuals with chronic HBV infection. HBV patients in Malaysia were found to have poorer knowledge compared to those in Singapore [19] and Chinese Canadians [21]. The majority of participants of this study were aware of the three significant complications of chronic HBV infection, namely chronic inflammation of liver, cirrhosis and liver cancer. However, participants were less aware of the symptoms of liver disease. While more than half of the respondents were able to respond correctly to two of the items (jaundice and tiredness), awareness of other symptoms such as nausea and vomiting, abdominal discomfort/pain and abdominal distension were less known. Studies have also shown that many HBV patients did not know that chronic HBV can be asymptomatic and may pass on the virus [20], and even would remain asymptomatic when the disease became much more severe or in early diseases such as liver cancer [19,21]. Sometimes the symptoms may be mistaken for other minor medical health conditions such as indigestion and are usually treated with over-the-counter medication. This is of concern as those who experience these symptoms may not be aware of the severity of the condition and thus may cause delay in seeking treatment. Early detection of the complication of HBV is important so that appropriate treatment can be given before the disease progresses. In addition, patients should also be made aware that cirrhosis and liver cancer are usually asymptomatic.

The misperception that sharing eating and drinking utensils could transmit HBV seemed to prevail among patients of this study. This misperception is also reported in Asian HBV patients [18,19,21] and general public in Singapore, San Francisco, Washington and Australia [23-25]. Such misperceptions could evoke anxiety in individuals with HBV and may create a barrier in their social interaction for fear of transmitting the virus to others. In addition, patients with HBV were found to be apprehensive about disclosing their disease status for fear of social rejection and yet they feel guilty about hiding their health condition [26]. This may hinder the timely diagnosis and treatment of the disease and its complications [26].

This study identified patients of age group 30–39, with tertiary education level and longer duration of HBV diagnosis as independent factors contributing to a higher knowledge score. Younger individuals have better opportunity to be educated and also have better access to information especially through the internet. Besides this, people with a high education level have higher health literacy which makes understanding of health information easier. In many studies, high educational level attainment has been reported to be associated with better HBV knowledge [7,11,19]. Individuals equipped with knowledge and education have been shown to be more likely to practice preventive measures such as getting screened for HBV and getting their children vaccinated for HBV [27]. Having longer duration of HBV diagnosis was also found to be associated with better knowledge of HBV. Having lived with HBV infection and in regular contact with healthcare professionals enable the patients to seek more information with regard to HBV. Perhaps this also explains why patients of aged 50–59 in this study have high knowledge. Patients without cirrhosis were found to be more knowledgeable with regards to hepatitis B. Perhaps, these patients were diagnosed with HBV early and equipped themselves with HBV knowledge and undertook necessary precautions to prevent the development to cirrhosis.

### Attitudes towards HBV

Majority of study participants were concerned of spreading HBV to family members and friends. In addition, most patients also showed positive attitudes regarding disclosure of disease status to family and health care providers. The reason could be that they are worried of their family getting infected as well especially from them. By informing their family members of the disease status, steps can be taken by their family members to go for screening which could lead to early diagnosis and treatment or vaccination for prevention. Besides, informing family members and healthcare providers could also enable both parties to take necessary precaution to avoid virus transmission since family members could be living with the patient while and healthcare providers have close proximity with patients when giving out health treatment which could increase the risk of transmission.

In contrast to disclosure of their HBV disease status to family and healthcare providers, participants reported feeling embarrassed to reveal their disease status to the public. This may indicate that there is an attached stigma to being HBV positive. Perhaps there are fears of social rejection. A qualitative study revealed that fear of rejection and stigma at personal and community level is a barrier for those with HBV to disclose their disease status as the revelation would lead to segregation from family and community [28].

Participants in this study were also found to be not empathetic towards people living with HBV infection. A third agreed that people with HBV should not be working in the food industry. This may be attributed to the misconception that HBV could spread by food. Demystifying these beliefs is necessary to prevent social isolation of and discrimination against people with HBV infection. These myths could be dispelled by patient and public health education.

Patients who reported feeling worried of spreading HBV to family and friends and worried since the diagnosis of HBV were of younger age group, Malay ethnicity, and had shorter duration of diagnosis. Reasons could be that HBV patients of younger age or those more recently diagnosed are worried as they may not have come to terms with the diagnosis of HBV infection and also were unsure of the characteristics of the disease, which explains the fear of transmission to others. While for some patients who might be knowledgeable, the worry may be stemmed from the knowledge of the consequences of long standing infection.

HBV patients were more likely to worry about spreading HBV infection to family and friends and worry ever since the diagnosis with increasing age. For those who received therapy for HBV, they were less likely to be worried after diagnosis. Early intervention is crucial for HBV patient just recently diagnosed such as in educating HBV patients with regards to their disease and also in initiating therapy for those who are eligible in order to prevent fears or anxiety in them.

### Lifestyle practices related to HBV prevention

With regards to lifestyle practices and HBV prevention, participants modified their lifestyles and adopted activities that would prevent virus transmission to others. The majority of patients undertook the necessary preventive measures to reduce the risk of HBV transmission, namely avoid sharing of personal items such as razors and toothbrushes, and also not participating in blood donation. In addition, they have also encouraged family members to screen for HBV. All the Indians who reported ever drinking alcohol had reduced or stopped drinking. However, it should be noted that there are only total of 3 Indians reported in this study who have ever drink alcohol. The small sample size of Indian in this study reflected the low prevalence of HBV among Indians in Malaysia due to lower genetic susceptibility to HBV as they originate from Southern India [29]. High proportion of Chinese also reported stopped or reduced drinking alcohol following their HBV diagnosis.

Approximately half or more of the participants avoided sharing eating and drinking utensils. This may stem from the misconception that sharing eating and drinking utensils could infect others. Those who are elderly, of Indian ethnicity and with cirrhosis were significantly found to not share their eating and drinking utensils. Older age groups may be less educated due to lack of education opportunity, understanding and access to health information. Most patients of Indian ethnicity who did not share eating and drinking utensils could be due to them worried of spreading HBV to their family and friends. This may be due to erroneous knowledge on viral transmission, which caused them to be worried of spreading to others and thus, adopting unnecessary behavior such as avoid sharing eating and drinking utensils. This finding highlighted the need for specific education intervention to be made targeted particularly to those of Indian ethnicity. Those with cirrhosis were also not sharing eating and drinking utensils. This again could be due to erroneous knowledge on viral transmission and especially when the symptoms of cirrhosis are much more obvious, thus may caused apprehension of both patient and others to share eating and drinking utensils with each other. Therefore, there needs to be more public health campaign to focus on the route of transmission of HBV.

This study found that patients with higher HBV knowledge were worried ever since diagnosed with HBV. In contrast to the logical assumption that people with higher knowledge should be less worried. Perhaps patient of this study, who felt worried ever since diagnosis sought more information with regards to the diseases and hence, more knowledgeable. However, studies have shown that knowledge does not necessarily enhance attitude and behavior in healthcare.

In Malaysia, nationwide campaigns are conducted to provide screening for hepatitis B, vaccination, and public forums to increase public awareness of the disease. Mass hepatitis B screening and vaccination have been found to decrease social stigma surrounding HBV [30]. A survey was carried out in 2000 in Malaysia to assess the effectiveness of Hepatitis Day and it revealed that although awareness of hepatitis is high in the public (95%), but it is only higher among those with higher level of education and income [30]. Perhaps different strategies can be employed to reach out to those from lower educational and socio economic background in terms of creating awareness and making preventive and treatment options more accessible. While this campaign has contributed greatly to increasing the awareness of HBV, in terms of reaching its target to educate public on symptoms, there is still room for improvement as evidenced by the findings from the current study. Hence, dissemination of information particularly on symptoms and modes of transmission of HBV needs to be intensified as knowledge pertaining to these two aspects are still lacking. This is pertinent as misconceptions on modes of transmission could potentially lead to stigma, unnecessary actions which would disrupt daily living and overall quality of life.

This study adds to the scanty research of Hepatitis B in the local context. However, this study was conducted in a hospital setting and may not be generalized to the wider population in the community.

## Conclusion

This study shows that HBV-related stigma and misconceptions are common among Malaysian patients with HBV. This is observed particularly in younger patients who have been diagnosed more recently. Therefore, health education, focussing on modes of transmission and symptoms of liver disease, should be targeted at these individuals. Majority of patients are worried about their disease and this calls for the healthcare professionals to explore and address patients’ concerns during clinical consultation. More research should be done to evaluate the effectiveness of public campaigns improving knowledge and demystifying HBV infection among the general public.

## Competing interests

The authors declare that they have no competing interests.

## Authors’ contribution

RM, conceptualization, conduct of the study, data analysis, manuscript write up. CJN, conceptualization, conduct of the study, data analysis, manuscript write up. WTT, data analysis, manuscript write up. SZA, write up. LPW, conceptualization, conduct of the study, data analysis. WYL, conceptualization, conduct of the study, data analysis, manuscript write up. All authors read and approved the final manuscript.

## Pre-publication history

The pre-publication history for this paper can be accessed here:

http://www.biomedcentral.com/1471-2458/12/601/prepub
